# Topological morphogenesis of neuroepithelial organoids

**DOI:** 10.1038/s41567-022-01822-6

**Published:** 2022-11-21

**Authors:** Keisuke Ishihara, Arghyadip Mukherjee, Elena Gromberg, Jan Brugués, Elly M. Tanaka, Frank Jülicher

**Affiliations:** 1 Max Planck Institute of Molecular Cell Biology and Genetics (MPI-CBG), Dresden, Germany; 2 Max Planck Institute for the Physics of Complex Systems (MPI-PKS), Dresden, Germany; 3 Center for Systems Biology Dresden, Dresden, Germany; 4 Cluster of Excellence Physics of Life, TU Dresden, Dresden, Germany; 5 Research Institute of Molecular Pathology (IMP), Vienna, Austria; 6 Present Address: Department of Computational and Systems Biology, School of Medicine, University of Pittsburgh, Pittsburgh, PA USA; 7 Present Address: Laboratoire de physique de l’École Normale Supérieure, Paris, France

**Keywords:** Biological physics, Membrane biophysics

## Abstract

Animal organs exhibit complex topologies involving cavities and tubular networks, which underlie their form and function^1–3^. However, how topology emerges during the development of organ shape, or morphogenesis, remains elusive. Here we combine tissue reconstitution and quantitative microscopy to show that tissue topology and shape is governed by two distinct modes of topological transitions^4,5^. One mode involves the fusion of two separate epithelia and the other involves the fusion of two ends of the same epithelium. The morphological space is captured by a single control parameter that can be traced back to the relative rates of the two epithelial fusion modes. Finally, we identify a pharmacologically accessible pathway that regulates the frequency of two modes of epithelial fusion, and demonstrate the control of organoid topology and shape. The physical principles uncovered here provide fundamental insights into the self-organization of complex tissues^6^.

## Abstract (web summary)

During development, tissues with complex topology emerge from collections of cells with simple geometry. This process in neuroepithelial organoids is governed by two topologically distinct modes of epithelial fusion.

## Main

Tissue morphogenesis is the emergence of increasingly complex geometry and topology out of a simple group of cells^7^. The geometry of a tissue characterizes its size and shape, and its topology defines how different parts are connected and characterizes the organization of cavities and passages between them^2,8^. Fundamental morphogenetic processes proceed as a series of size and shape changes and topological transitions^3,6,9,10^. For example, gastrulation involves the invagination of an epithelial cell layer of initially spherical topology that eventually transitions to a toroidal topology serving as the precursor for the passage connecting the mouth to the other end^1^. In the case of vasculogenesis, endothelial tissues form tubular geometries that connect and result in complex topological networks with branches and loops^11^. Fluid-filled cavities called lumens form the basis of transport networks such as bile canaliculi in the liver^12^ and ventricles in the brain^13^. Pathological conditions such as polycystic kidney disease are associated with altered organ topology^14^. Thus, topological and geometric transitions play a key role during morphogenesis and in organ function. However, the principles that guide the interplay of topology and geometry in formation of complex organ architectures remain unknown.

**Fig. 1:**
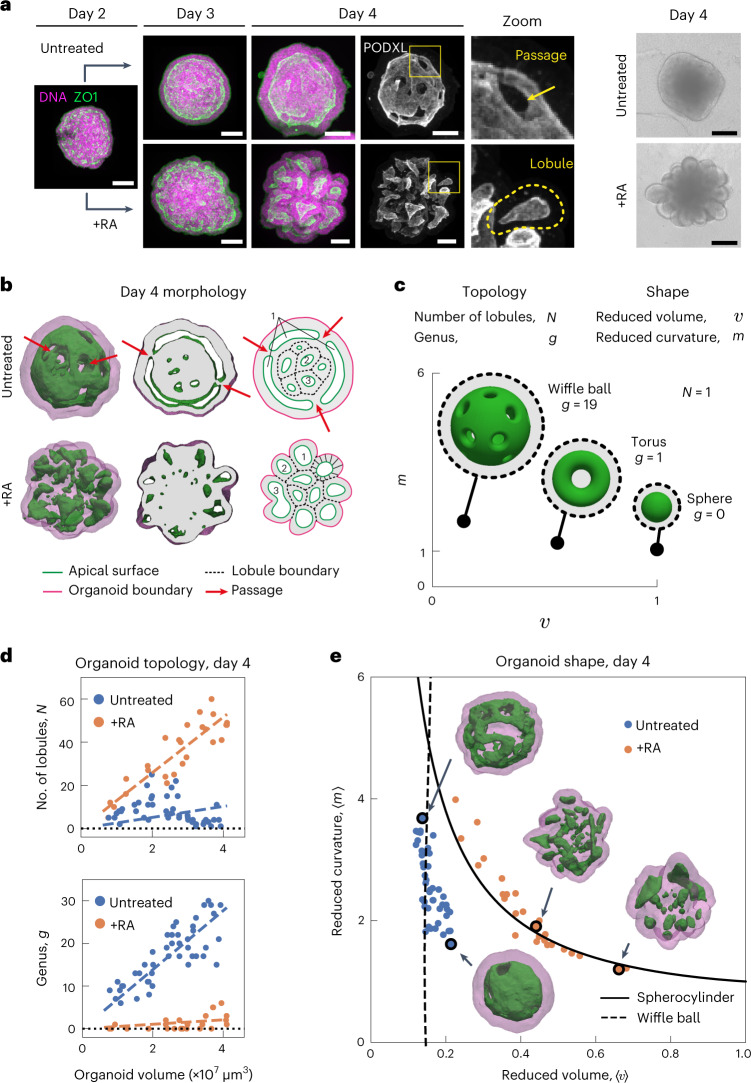
Topology and shape of neuroepithelial organoids. **a**, Neuroepithelial organoids with different morphologies were generated in the presence or absence of RA. Immunofluorescence for ZO1 (green) and PODXL (white) marks the apical membranes. DNA staining (magenta) marks the entire organoid. The images are the maximum intensity projections of volumetric images. Extended Data Fig. 1a shows the images of individual confocal slices. The example of passages on the apical surface (zoom, top row). Example of an epithelial lobule (zoom, bottom row). Bright-field images show the difference in the outer morphology of organoids in the two conditions. **b**, Organoid morphology is visualized by the surface renderings of the organoid outer boundary (magenta, transparent) and its apical surfaces (green) for day 4 immunostained samples in **a**. In the cross sections, the grey regions indicate the volume occupied by the cells, whereas the white regions indicate the fluid-filled lumens. The schematic shows how organoids are divided into epithelial lobules annotated with numbers 1, 2 and 3. Extended Data Fig. 1c shows a schematic of the cell-level interpretation of passages. **c**, Topology of an organoid is quantified by the number of epithelilal lobules *N* and total genus *g*. The shape of the organoid is quantified by the average reduced volume 〈*v*〉 and average reduced curvature 〈*m*〉, calculated from the values of all the epithelial lobules (Extended Data Fig. 2c, Methods and Supplementary Note). **d**, Topology of untreated (blue, *n* = 45) and RA-treated organoids (orange, *n* = 27) at day 4 are characterized by the number of epithelial lobules *N* and total genus *g*, and displayed with respect to the organoid volume. Organoids of different sizes were generated by varying the number of cells to be seeded at day 0 from 300 to 2,400 cells. The dashed lines are linear fits with zero intercept. **e**, Shape of untreated and RA-treated organoids are represented in the shape diagram, based on the average reduced volume 〈*v*〉 and average reduced curvature 〈*m*〉. The parametric curves for the wiffle ball (four passages, *d*/*R* = 0.15; dashed line) and spherocylinders (solid line) serve as guides. The morphology of the representative organoids (black circles) from both conditions are displayed. Extended Data Fig. 1e shows the shapes of individual lobules in these organoids. Scale bars, 100 μm. Source data

**Extended Data Fig. 1:**
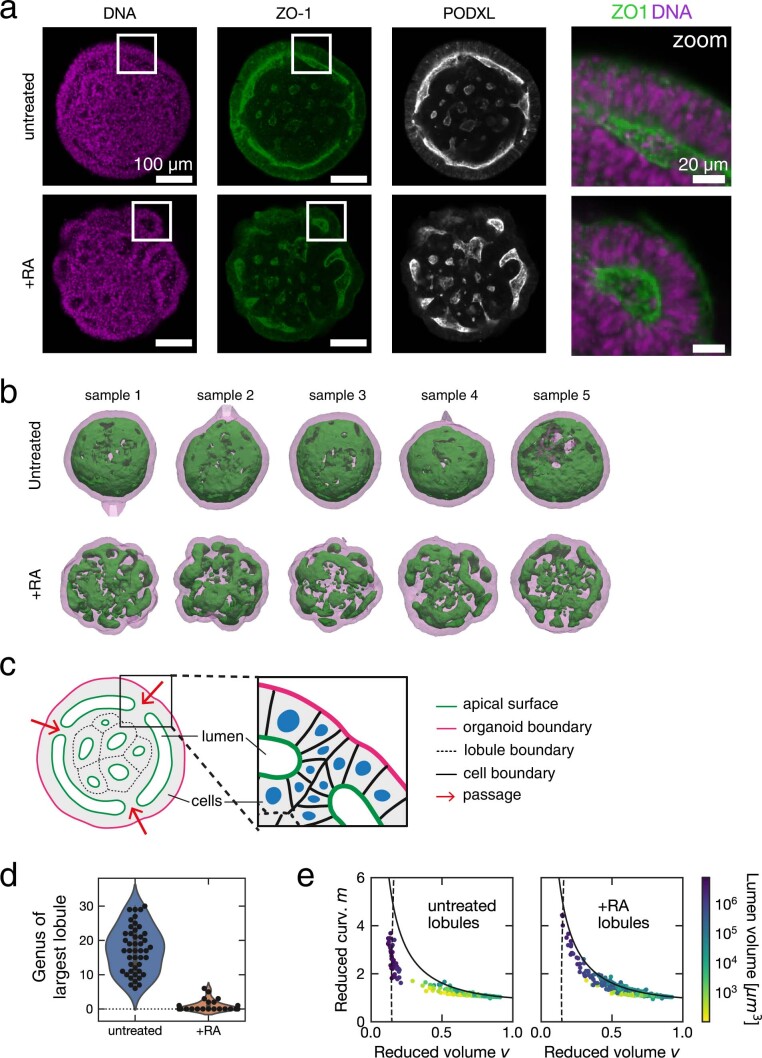
3D imaging and analysis of optically cleared organoids samples. **a**, Single planes from spinning disc confocal microscopy images of optically cleared organoids stained for DNA, anti-ZO1, and anti-PODXL. **b**, Examples of untreated and RA-treated organoids at Day 4. Surface representations show organoid outer boundary (magenta, transparent) and apical surfaces (green). **c**, Schematic representation of an organoid cross section, describing the relation between the organoid outer boundary (magenta), apical surfaces (green), lobule boundary (dotted lines), cell nuclei (blue), and passages (red arrow). **d**, Genus of the largest lobule in each organoid. **e**, Shape diagram of individual lobules found in multiple organoids. Lumen volume is indicated by colour. Solid line: spherocylinders. Dashed line: wiffle ball. Panels **b** through **c** show data from the same n=45 untreated and n=27 RA-treated organoids as in Fig. 1.

To address this fundamental issue, we make use of recent advances in three-dimensional (3D) organ reconstitution that provide accessible and controllable experimental systems to study how a simple assembly of cells dynamically organizes into tissues with complex architectures^15,16^. We differentiate mouse embryonic stem (ES) cells in vitro as free-floating aggregates that develop into neuroepithelial organoids in four days (Fig. 1a and Extended Data Fig. 1a)^17,18^. Staining of apical surfaces (anti-ZO1, anti-PODXL) suggests that this process involves the formation of fluid-filled lumens. The apical surface of these organoids contained many passages (Fig. 1a, top), indicating the emergence of complex tissue geometry and topology. Here, to explore organoid morphogenesis, we set out to quantify geometry and topology and use biochemical perturbations to influence geometric and topological changes. Retinoic acid (RA) is known to act as a morphogen and to instruct the size and shape of the developing neural tube^19^. We found that RA treatment at day 2 of differentiation had a substantial influence on the geometry and topology of organoids. Staining of apical surfaces revealed the emergence of multiple epithelial lobules by day 4 (Fig. 1a, zoomed-in view, bottom), in contrast to untreated organoids that are dominated by one large lobule (Fig. 1a, untreated).

**Extended Data Fig. 2:**
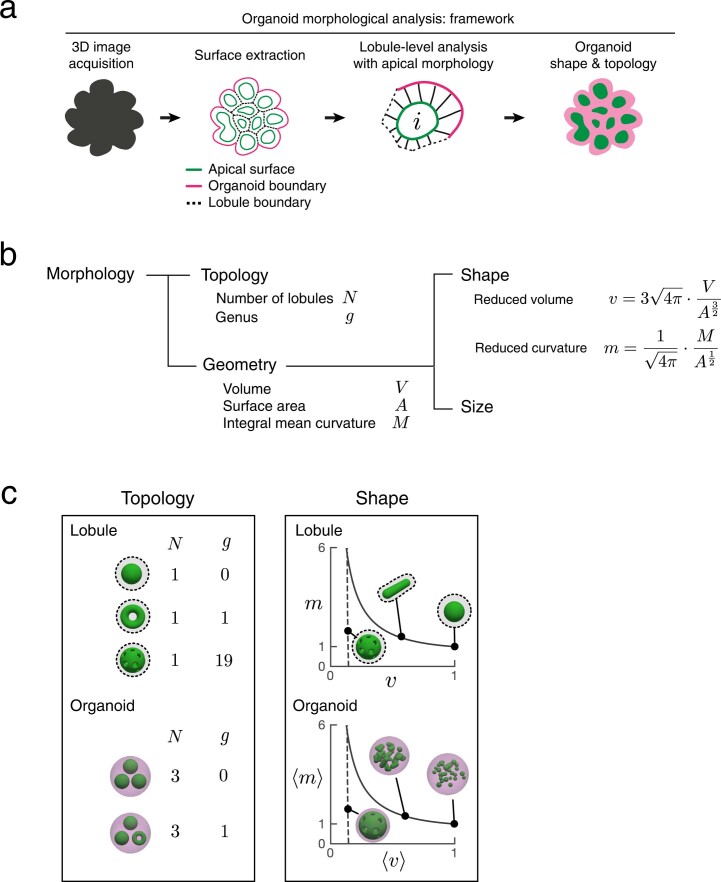
Framework for morphological analysis of organoids. First, we acquire 3D images of a tissue sample, here, an organoid. Second, we perform image segmentation and surface construction of triangulated meshes to represent 3D tissue morphology as a set of apical surfaces and organoid outer boundary. Next, we use individual apical surfaces to define epithelial lobules and characterize their morphology. Finally, we integrate the information from all lobules to define the overall topology and shape of an organoids. This process is repeated for multiple organoids. **b**, In mathematical terms, morphology is discussed for the aspects of topology and geometry. Topology describes the connectivity of objects, which do not change from stretching or shrinking. Geometry is discussed in terms of the shape and size of objects. Shape is a scale invariant quality, while size has the units related to length scale. **c**, The metrics for topology are the number of lobules *N* and total genus *g* in an organoid. The metrics for shape are the reduced volume *v* and reduced curvature *m* of individual lobules, which are averaged to yield organoid level quantities 〈*v*〉 and 〈*m*〉 (see Methods).

To quantify organoid geometry and topology, we segmented the organoid architecture and constructed triangular meshes to define the outer (magenta) and apical surfaces (green) (Fig. 1b, Methods, Supplementary Videos 1 and 2, and Extended Data Fig. 1b). This allowed us to quantify key geometric and topological measures for each organoid (Extended Data Fig. 2a,b). Using the apical surface to define distinct epithelial lobules, we characterized organoid topology by the number of lobules *N* and their topological genus *g*, and the shape of each lobule by its reduced volume *v* and reduced curvature *m* (Fig. 1c and Supplementary Note). Organoid shape is then characterized by the average of the reduced volumes and reduced curvatures over all the lobules, 〈*v*〉 and 〈*m*〉, respectively (Methods and Extended Data Fig. 2c). We observed that untreated organoids contained large lobules with high genus *g* that increased with organoid volume (Fig. 1d and Extended Data Fig. 1d), with shape and topology that can be qualitatively captured by the wiffle ball morphology (compare with Fig. 1b,c, inset). In contrast, RA-treated organoids contained lobules of spherical topology (*g* = 0), with lobule number *N* increasing with organoid volume (Fig. 1d and Extended Data Fig. 1d).

The geometry of organoids can be represented in a shape diagram (〈*v*〉, 〈*m*〉) (refs. ^20–22^) (Fig. 1e). Untreated organoids at day 4 (blue points) had similar average reduced volumes 〈*v*〉 around 0.15, but varying in average reduced curvature 〈*m*〉 falling approximately on a vertical line (Fig. 1e, dashed line). This line corresponds to wiffle ball configurations for varying passage sizes (Supplementary Note). For RA-treated organoids at day 4 (Fig. 1e, orange points), the average reduced volume 〈*v*〉 decreases for increasing average reduced curvature 〈*m*〉. This trend falls on the line (solid) corresponding to spherocylinder shapes of varying aspect ratios, ranging from spheres to tubes (Fig. 1e and Supplementary Note). Thus, by day 4, untreated organoids develop into a morphology dominated by a large lobule of high genus resembling a wiffle ball, whereas RA-treated organoids develop into many lobules of low topological genus consisting of spherocylinders.

**Fig. 2:**
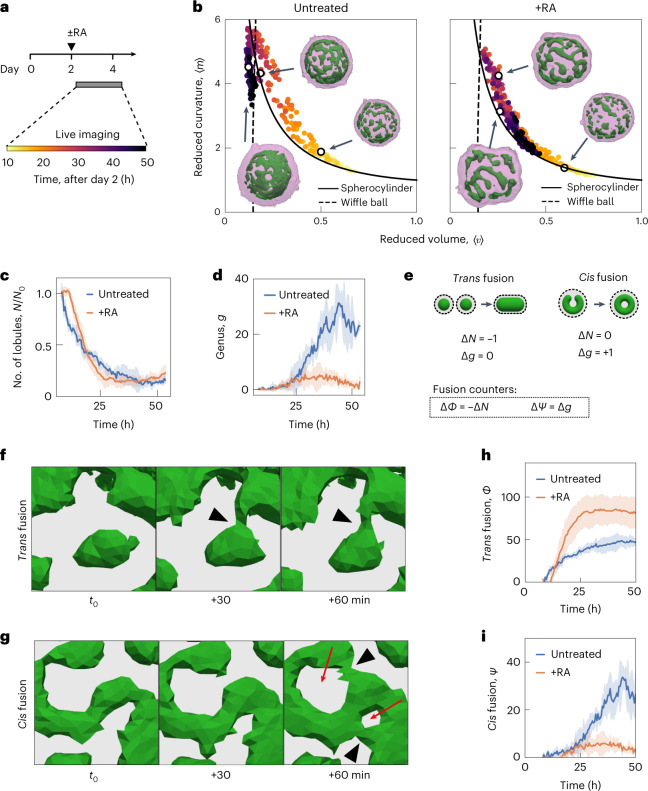
*Trans* and *cis* modes of epithelial fusion underlie the emergence of organoid topology and shape. **a**, Experimental timeline for the live imaging experiment to observe organoid morphogenesis. For each time point, 3D images of SiR-actin were used to segment and reconstruct the surfaces that represent the tissue outer boundary (magenta) and apical membranes (green). **b**, Organoid shape trajectory from day 2 to 4 for *n* = 3 untreated organoids (left) and *n* = 4 RA-treated organoids (right). The parametric curves for the wiffle ball (four passages, *d*/*R* = 0.15; dashed line) and spherocylinders (solid line) serve as guides. Time indicates hours after RA treatment on day 2. Extended Data Fig. 3b shows the shape trajectory of the largest lobule in the organoids. **c**, Temporal change in the number of lobules *N* normalized to the beginning of the video for each organoid. Extended Data Fig. 3c shows the number of lobules *N*. **d**, Temporal change in the total genus *g* of organoids. Extended Data Fig. 3d shows the temporal change in the genus of the largest lobules. **e**, *Trans* fusion and *cis* fusion are two distinct modes of topological transitions that underlie organoid morphogenesis. We define the counting variables for *trans* and *cis* fusion solely based on the changes in topological *N* and *g*. The schematic shows the typical geometric changes for epithelial fusion observed in our experiments. **f**, Example of *trans* fusion in which two separate epithelial lobules fuse their apical surfaces (green). **g**, Example of *cis* fusion in which a single epithelial lobule (apical surface, green) fuses with itself. The grey regions indicate the volume occupied by the cells. The arrowheads indicate the location of fusion. The red arrows indicate newly formed passages. **h**, Cumulative number of *trans* fusion events *Φ* over time. **i**, Cumulative number of *cis* fusion events *Ψ* over time. For **c**, **d**, **h** and **i**, the error bars indicate the standard deviation of *n* = 3 untreated and *n* = 4 RA-treated organoids. Source data

**Extended Data Fig. 3:**
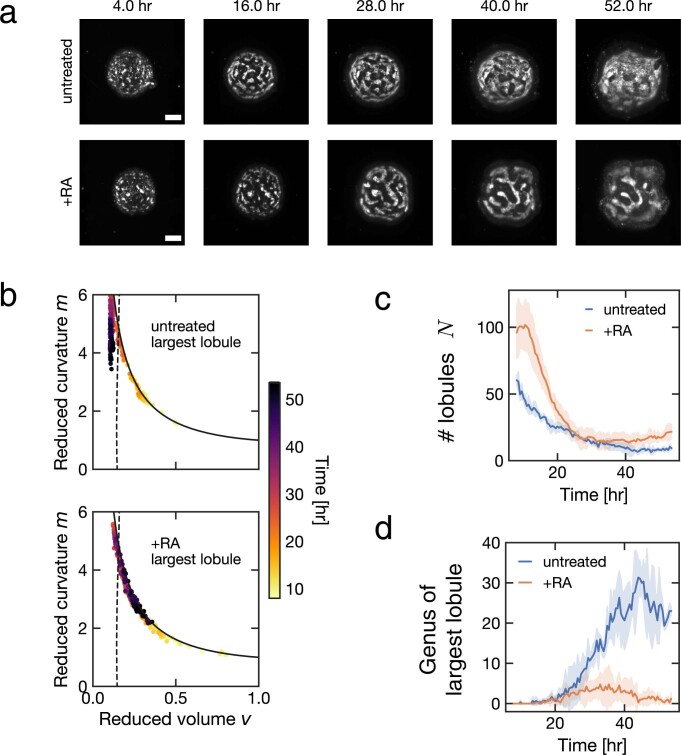
4D imaging of organoid morphogenesis. **a**, Individual frames from light sheet imaging experiment of organoids. Images shown are maximum intensity projections of SiR-actin signal. Time indicates time post RA treatment. **b**, Shape diagram displays how the shape of the largest lumen in the organoid evolves over time. Solid line: spherocylinder. Dashed line: wiffle ball. **c**, The temporal change in the number of lobules *N* in organoids. **d**, The temporal change in the genus *g* of the largest lobule of each organoid. Panels show data from the same n=3 untreated and n=4 RA-treated organoids as in Fig. 2. Scale bar, 100 μm.

To address how and when these two different organoid morphologies emerge, we imaged SiR-actin-labelled organoids using light sheet microscopy over 48 h from day 2 to 4 (Fig. 2a, Extended Data Fig. 3a, and Supplementary Videos 3 and 4). At the initial stages of imaging at day 2, organoids in both conditions contained numerous small spherical lobules, which fused with each other and gave rise to elongated and tubular lobules (Supplementary Videos 5 and 6 show the surface renderings). From day 2 to 3, the organoid shapes in both conditions followed a trajectory along the spherocylinder branch in the shape diagram, starting from elongated spheres (〈*v*〉 ≃ 1, 〈*m*〉 ≃ 1) and increasing in aspect ratio (Fig. 2b). At about 24 h of imaging (day 3), the organoid shapes reached a region in the shape diagram where the wiffle ball and spherocylinders morphologies meet (intersection of solid and dashed lines in Fig. 1e; (〈*v*〉 ≃ 0.15, 〈*m*〉 ≃ 5)). After 24 h, the shape trajectories for the two conditions diverged. Untreated organoids transitioned from the spherocylinder branch to the wiffle ball branch, decreasing the average reduced curvature with time and maintaining a small average reduced volume (Fig. 2b, left). In contrast, RA-treated organoids remained on the spherocylinder branch, but returned towards larger average reduced volumes by day 4 (Fig. 2b, right). During the two days of imaging, the number of lobules *N* decreased monotonically in a similar manner for both conditions (Fig. 2c and Extended Data Fig. 3c). The trajectories of the total genus *g* in both conditions remained low until about 24 h, after which they diverged. In the untreated condition, *g* increased over time to large values from day 3 to 4, whereas in the RA-treated condition, *g* remained small even at day 4 (Fig. 2d).

The time dependence of lobule number and total genus indicated that topological transitions occur during organoid morphogenesis from day 2 to 4. A close examination of the apical surface triangulations revealed that topological transitions occurred as two distinct modes of fusion processes irrespective of geometry. One mode of fusion, which we call *trans* fusion, involves two separate lobules that fuse with each other (Fig. 2e, left). This results in a reduction in lobule number by one (Δ*N* = −1). The second mode of fusion, which we call *cis* fusion, involves a single lobule that fuses with itself to create a passage (Fig. 2e, right). This results in an increase in genus by one (Δ*g* = +1). Experimentally observed examples of *trans* and *cis* fusion are shown in Fig. 2f,g, respectively, and a schematic is shown in Fig. 2e. We defined the counters for *trans* and *cis* fusion (Fig. 2e), and quantified the cumulative fusion events from day 2 to 4. In both conditions, the early time points are dominated by *trans* fusion events (Fig. 2h), whereas beyond the 24 h time point, *cis* fusion is the primary mode of fusion observed only for untreated organoids (Fig. 2i). Thus, we discovered that organoid topologies emerge from distinct modes of fusion, namely, *trans* and *cis* fusion, where only the latter leads to the creation of passages and epithelial tissues with non-spherical topology.

**Fig. 3:**
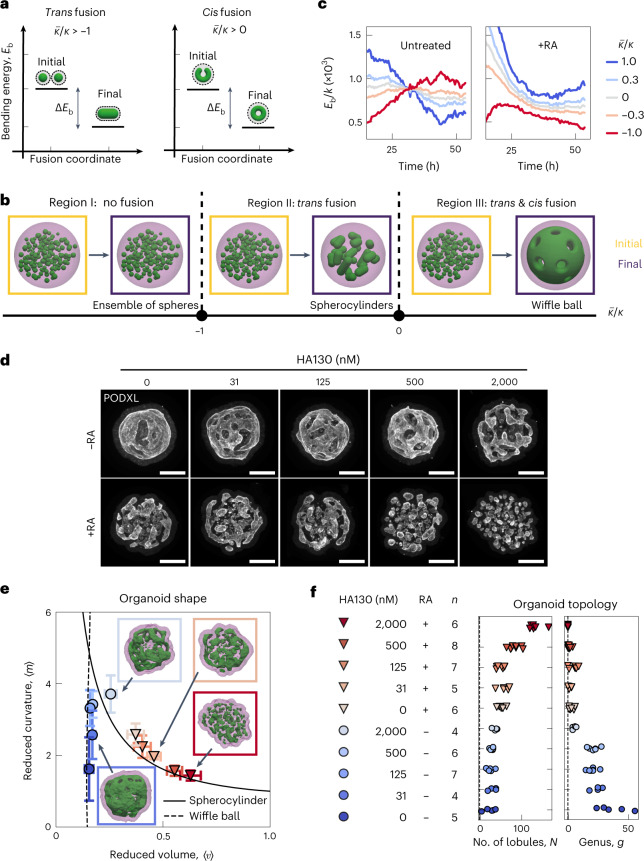
Energetics of *trans* and *cis* fusion and pharmacological control of organoid morphogenesis. **a**, Change in bending energy Δ*E*_b_ is depicted for *trans* and *cis* fusion. The conditions for Δ*E*_b_ < 0 is denoted for both fusion modes in terms of topological rigidity $\bar{\kappa }/\kappa$. **b**, State diagram of organoid morphology is shown as a function of reduced Gaussian rigidity $\bar{\kappa }/\kappa$. Three regions depict the initial (yellow) and final (purple) morphologies. Region I, $\bar{\kappa }/\kappa < -1$: an ensemble of spheres is stable and no fusion is favoured. Region II, $\bar{\kappa }/\kappa > -1$: *trans* fusion leads to the formation of tubular lobules with spherical topology. Region III, $\bar{\kappa }/\kappa > 0$: both *trans* and *cis* fusion are favoured and leads to lobules with wiffle ball morphology. **c**, Temporal evolution of scaled bending energy is shown for various values of $\bar{\kappa }/\kappa$, estimated from triangulated meshes ${E}_{\mathrm{b}}/\kappa =\sum {H}_{i}^{2}{A}_{i}+4\uppi (\bar{\kappa }/\kappa )(N-g)$, where index *i* traverses all the faces in the system. Extended Data Fig. 4a shows the evolution of individual terms and Extended Data Fig. 4b shows the corresponding energy for the largest lobule in the system. Estimations were made for the same *n* = 3 untreated and *n* = 4 RA-treated organoids represented in Fig. 2. **d**, Treatment with HA130 leads to organoid morphologies that are consistent with the attenuation of epithelial fusion, both in the absence (top row) and presence (bottom row) of RA. Supplementary Videos 11 and 12 show the surface rendering examples. **e**, Shape of organoids cultured in varying concentrations of HA130. The error bar indicates 95% confidence interval for each condition (**f** shows the legend and *n* organoids). Organoid morphology is visualized by the surface renderings of the organoid outer boundary (magenta, transparent) and its apical surfaces (green) for day 4 samples shown in **d**. The parametric curves for the wiffle ball (four passages, *d*/*R* = 0.15; dashed line) and spherocylinders (solid line) serve as guides for the shape diagram. **f**, Topology of organoids cultured in varying concentrations of HA130. Source data

**Extended Data Fig. 4:**
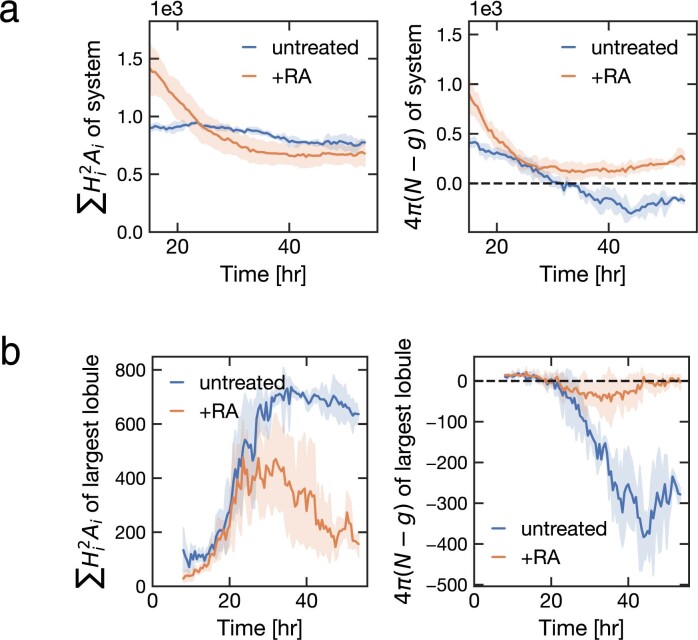
Energetics of epithelial morphogenesis. **a**, Temporal evolution of bending elasticity (${\sum }_{i}{H}_{i}^{2}{A}_{i}$) and Gaussian elasticity (∫*KdA* = 4*π*(*N* − *g*)) is depicted for organoids. **b**, Temporal evolution of bending elasticity (${\sum }_{i}{H}_{i}^{2}{A}_{i}$) and Gaussian elasticity (∫*KdA* = 4*π*(*N* − *g*)) is depicted for largest epithelial lobules of each organoids. Errorbars indicate the standard deviation of the same n=3 untreated and n=4 RA-treated organoids represented in Fig. 2.

These findings suggest that the topology and shape of the emerging tissue are determined by the *trans* and *cis* modes of topological transitions that are generated during morphogenesis. This raises the question of which epithelial properties determine the dominant mode of fusion and how they are controlled. One possibility is that tissue mechanics^23–25^ governs the fusion events and favours one mode of fusion over the other. An example of a mechanical property that distinguishes between the *trans* and *cis* modes of fusion is the bending energy of a fluid surface^21,26–29^

1 $${E}_{\mathrm{b}}=\int [\kappa {H}^{2}+\bar{\kappa }K]\mathrm{d}A,$$

where *H* denotes the local mean curvature of the surface, *K* denotes the Gaussian curvature and d*A* is the area element. For fluidized epithelial surfaces, the bending rigidities can be estimated from cell-based physical models of tissue mechanics^30–34^ (Supplementary Note). The bending rigidity *κ* and Gaussian rigidity $\bar{\kappa }$ are elastic moduli that describe the resistance of the shape to bending and saddle-splay deformations, respectively. Note that ∫*K*d*A* = 4π(*N* − *g*) only depends on the indices *N* and *g* counting the lobules and passages, respectively. Therefore, Gaussian rigidity $\bar{\kappa }$ describes the resistance to topological changes. In addition, bending rigidity *κ* not only governs changes in shape, but also changes in lobule number *N*. We discuss how $\bar{\kappa }$ and *κ* could affect the mode of fusion by comparing bending energy *E*_b_ before and after fusion (Fig. 3a and Supplementary Note). Briefly, the change in *E*_b_ associated with *trans* fusion of two lobules (Fig. 3a, left) is ${{\Delta }}{E}_{\mathrm{b}}\simeq -4\uppi (\kappa +\bar{\kappa })$; thus, *trans* fusion is energetically favoured when $\kappa +\bar{\kappa } > 0$. The change in *E*_b_ for *cis* fusion is ${{\Delta }}{E}_{\mathrm{b}}\simeq -4\uppi \bar{\kappa }$; thus, passage formation via *cis* fusion in a lobule is energetically favoured when $\bar{\kappa } > 0$ (Fig. 3a, right). If $\bar{\kappa } < -\kappa$, neither *trans* or *cis* fusion is energetically favoured. These criteria can be summarized in a state diagram as a function of the ratio $\bar{\kappa }/\kappa$ of the two elastic moduli, which is the reduced Gaussian rigidity. We can distinguish three parameter regions, where morphologies evolve differently when starting from an initial state of *N* spherical lobules (Fig. 3b). In region I, $\bar{\kappa }/\kappa < -1$ and fusion is energetically disfavoured. The system remains in a configuration with many spherical lobules (large *N*, *g* = 0). In region II, $-1 < \bar{\kappa }/\kappa < 0$ and only *trans* fusion is energetically favoured. In this case, lobules will tend to fuse, resulting in fewer lobules of spherical topology (small *N*, *g* = 0). In region III, $0 < \bar{\kappa }/\kappa$, both *trans* and *cis* fusion are energetically favoured. As a result, large lobules with many passages exemplified by wiffle ball morphology emerge (small *N*, large *g*). Our results suggest that RA treatment shifts the behaviour of the system from region III to region II. To test this idea, we estimate the scaled bending energy *E*_b_/*κ* of lobule geometries as a function of time (Supplementary Videos 7, 8, 9 and 10) considering different values of reduced Gaussian rigidity $\bar{\kappa }/\kappa$ (Methods, Fig. 3c (left) and Extended Data Fig. 4). Only for positive $\bar{\kappa }/\kappa$ corresponding to region III, scaled bending energy *E*_b_/*κ* decreases with time for untreated organoids, consistent with the idea that untreated organoids operate in region III. After RA treatment, a decrease in scaled bending energy *E*_b_/*κ* with time requires a shift in $\bar{\kappa }/\kappa$ to negative values (Fig. 3c, right). This suggests that after RA treatment, the mechanical properties of the epithelia is modified, possibly leading to a reduction in $\bar{\kappa }/\kappa$ below zero at later times.

**Extended Data Fig. 5:**
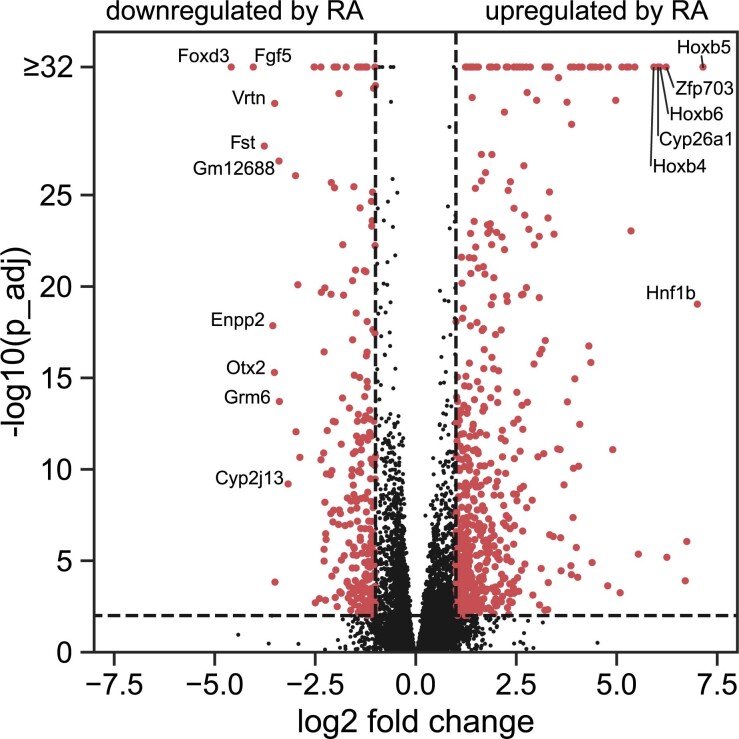
Gene expression changes in response to retinoic acid. Volcano plot displaying gene expression change in organoids treated with or without retinoic acid (RA). Red points represent all differentially regulated genes (see Methods and Table S1 for a complete list).

**Extended Data Fig. 6:**
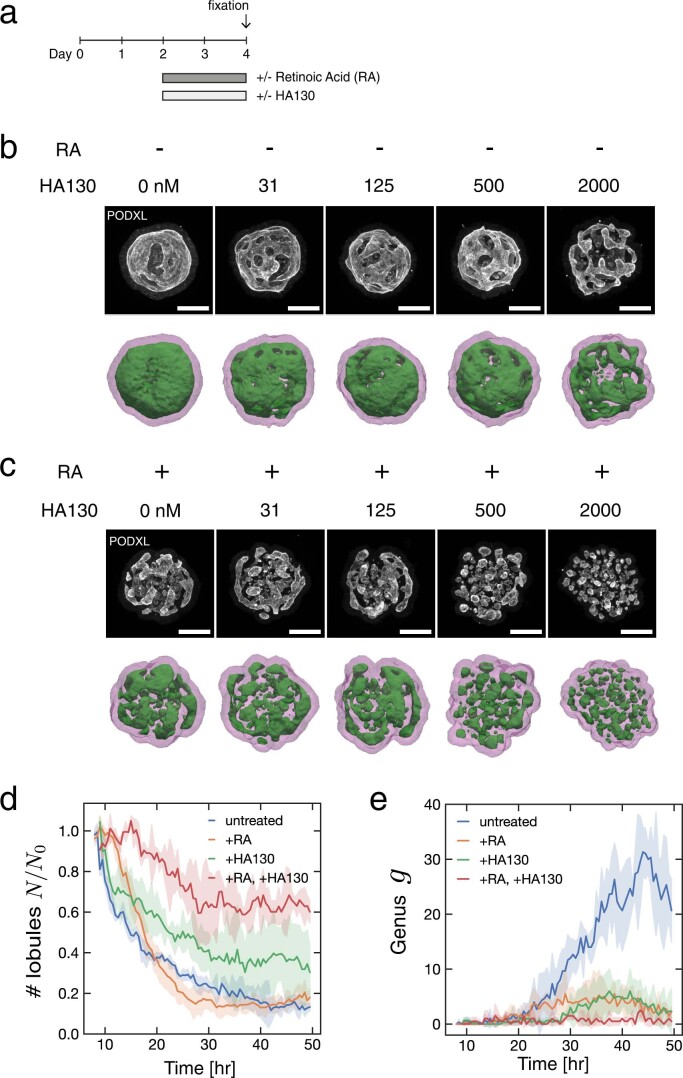
Lysophosphatidic acid pathway and pharmacological control of topological morphogenesis. **a**, Workflow of experiment to test the role of lysophosphatidic acid (LPA) synthesis in organoid morphogenesis. Organoids were treated with varying concentrations of HA130, a small molecular inhibitor for ENPP2, with or without 250 nM retinoic acid (RA) during Day 2 to Day 4. Organoid morphologies were scored at Day 4. **b**, Representative examples of Day 4 organoids treated with varying concentrations of HA130 in the absence of RA. **c**, Representative examples of Day 4 organoids treated with varying concentrations of HA130 in the presence of RA. Images show maximum intensity projections of PODXL immunofluorescence signal. Organoid outer boundary (magenta) and apical surfaces (green) are shown for the corresponding sample. **d**, The temporal change in the number of lobules *N* normalized to the beginning of the movie for each organoid under the combination of RA and HA130 treatments. **e**, The temporal change in the total genus *g* of organoids under the combination of RA and HA130 treatments. The conditions +HA130 (n=3 organoids treated with 2000 nM HA130) and +RA, +HA130 (n=2 organoids treated with 250 nM RA and 2000 nM HA130) are presented together with the data for untreated and +RA conditions from Fig. 2. Scale bar, 100 *μ*m.

**Extended Data Fig. 7:**
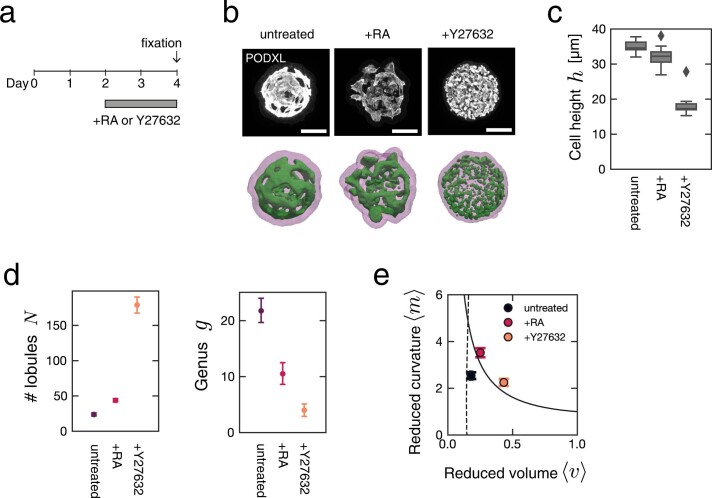
Cell contractility and mechanics underlie topological morphogenesis. **a**, Workflow of experiment to test the role of cell contractility in organoid morphogenesis. Organoids were treated with 250 nM retinoic acid (RA) or with 20 *μ*M of Y-27632, a small molecular inhibitor for ROCK kinase during Day 2 to Day 4. Organoid morphologies were scored at Day 4. **b**, Representative examples of Day 4 organoids treated with RA or Y-27632. Images show maximum intensity projections of PODXL immunofluorescence signal. Organoid outer boundary (magenta) and apical surfaces (green) are shown for the corresponding sample. **c**, Measurement of epithelial cell height based on the distance between the apical surfaces and the outer organoid boundary. **d**, Topology of organoids. **e**, Shape of organoids. Parametric curves for the wiffle ball (4 passages, *d*/*R* = 0.15, dashed line) and spherocylinders (solid line) serve as guides for the shape diagram. Errorbar indicates 95% confidence interval from n=12, 18, 9 organoids for the untreated, +RA, +Y27632 conditions, respectively. Scale bar, 100 *μ*m. Source data

Thus, the primary effect of RA treatment on topological morphogenesis is consistent with a decrease in the reduced Gaussian rigidity $\bar{\kappa }/\kappa$. This raises the question whether we can tune the relative rates of *trans* and *cis* fusion via further molecular perturbations and control the topological morphogenesis. To identify such a molecular pathway, we performed RNA sequencing (RNAseq) on organoids treated with or without RA (Methods). RA treatment led to a broad change in the expression of developmental genes (Supplementary Table 1 and Extended Data Fig. 5) consistent with previous work^35,36^. We found that RA treatment downregulated the expression of *Enpp2*, which encodes the cellular enzyme that produces lysophosphatidic acid (LPA)^37^. LPA is known to enhance the folding of developing mouse brains^38^ and increase the apical membrane area in human^39^ and gorilla^40^ neuroepithelial cells. To test the role of LPA synthesis on organoid morphogenesis, we treated day 2 organoids with varying concentrations of HA130, a small molecule inhibitor of LPA synthesis^41^, and analysed their morphology at day 4 (Fig. 3d (top) and Extended Data Fig. 6a,b). At low concentrations of HA130 (31–125 nM), the organoids primarily had a single lobule with many passages, resembling wiffle ball morphologies, similar to the untreated case and consistent with region III (Fig. 3e,f). Increasing the concentration of HA130 resulted in morphologies with fewer passages until at the highest concentration of HA130 used (2000 nM), the organoids resembled a set of elongated spheres, consistent with the gradual shift from region III to region II (Fig. 3d (top right) and Supplementary Video 11). This perturbation shows that the inhibition of LPA synthesis mimics the effect of RA treatment in organoid morphogenesis. Our theory based on reduced Gaussian rigidity predicts that further reduction in $\bar{\kappa }/\kappa$ can result in a transition from region II to region I, where both *trans* and *cis* fusion are disfavoured. This behaviour was not originally observed in the presence or absence of RA. To test this prediction, we applied varying concentrations of HA130 to RA-treated organoids, thus combining the effect of RA treatment and inhibition of LPA synthesis (Fig. 3d (bottom) and Extended Data Fig. 6a,c). For increasing concentrations of HA130 (31–2,000 nM), RA-treated organoids exhibited increasing numbers of lobules (Fig. 3f), consistent with the gradual suppression of *trans* fusion. At the highest concentrations of HA130 (500 and 2,000 nM), RA-treated organoids resulted in an ensemble of spherical lobules with lobule number *N* at day 4 comparable with day 2 organoids (Fig. 3d (bottom right) and Supplementary Video 12). This is consistent with the suppression of *trans* and *cis* fusion and a shift to region I in the state diagram (Fig. 3b). To follow the changes in topology for these conditions, we performed live imaging experiments and found that 2,000 nM HA130 greatly reduced the rates of *trans* and *cis* fusion (Extended Data Fig. 6d,e). By combining the effects of RA treatment with HA130, we achieved an attenuation of topological transitions corresponding to a shift in organoid morphogenesis from region III via region II to region I. The corresponding organoid shapes followed a trajectory in the (〈*v*〉, 〈*m*〉) diagram starting from the wiffle ball branch (Fig. 3e, dashed) at low 〈*m*〉, moving upwards and shifting towards region II. The trajectory transitions to the spherocylinder branch (Fig. 3e, solid) when passages are no longer observed corresponding to region II. The trajectory follows this branch towards large 〈*v*〉 and small 〈*m*〉 as the system shifts to region I. At the cellular level, the treatment with RA and HA130 lead to the reduced production of LPA, which is known to increase cortical contractility via the Rho/ROCK/myosin pathway^42^. To directly test the role of cell contractility on topological morphogenesis, we treated organoids with the ROCK kinase inhibitor Y-27632, which led to organoid topology and shape consistent with the prediction of reduced $\bar{\kappa }/\kappa$ and attenuation of fusion (Extended Data Fig. 7 and Supplementary Note). Note, however, that changes in cell mechanics may have consequences beyond Gaussian and bending rigidity. These pharmacological modulations allowed us to tune the topological morphogenesis by controlling the modes of fusion and their rates, giving rise to a broad range of morphologies with varying shapes and topologies.

In summary, by combining quantitative four-dimensional microscopy, theory and pharmacological perturbations, we have discovered how topological transitions drive neuroepithelial morphogenesis through *trans* and *cis* epithelial fusion. The morphological space can be captured by a single control parameter of epithelial fusion rates. We propose that this parameter is governed by the reduced Gaussian rigidity $\bar{\kappa }/\kappa$ of an epithelial surface. The physical principle of topological morphogenesis could apply to organ development in diverse contexts^43,44^. Topological morphogenesis may explain the normal development and pathology of the posterior neural tube, in which loops^45,46^ and multiple ectopic tubes^47,48^ have been reported. In engineering applications, we envision that the control of tissue topology will guide new strategies to design synthetic tissues.

## Methods

### Mouse ES cell and neuroepithelial organoid culture

Mouse ES cells were passaged in N2B27 medium with 2i/LIF following standard protocols^49^. To generate neuroepithelial organoids, we modified a published protocol for optic cup organoids^17^. On day 0, the ES cells were dissociated into single cells and re-aggregated in N2B27 neural induction medium (typically 1,000 cells per 50 μl per ml per well) in 96-well U-bottom low adhesion plates (ThermoFisher, 174925). On day 1, Matrigel (BD Biosciences, 354234) dissolved in 50 μl N2B27 medium was added to each well for a final concentration of 2.5% (v/v). On day 2, 50 μl N2B27 with or without all-*trans* RA (Sigma R2625) was added to each well. The final concentration of RA was 250 nM. In some experiments, organoids were treated with varying concentrations of HA130 (Echelon Biosciences, B-0701) to inhibit LPA synthesis, or with 20 μM Y-27632 (Tocris, #1254) to inhibit ROCK kinase. All the 3D morphological data in this study were collected from neuroepithelial organoids made from E14 mouse ES cells (a gift from M. Shahbazi, MRC Laboratory of Molecular Biology). The major morphological phenotypes induced by the addition of RA and HA130 were confirmed by experiments using R1 wild-type mouse ES cells (a gift from R. Naumann, MPI-CBG).

### Immunofluorescence and optical clearing of organoids

The organoid samples were washed in phosphate-buffered saline (PBS), fixed with ice cold 4% paraformaldehyde solution for 30 min, rinsed and stored in PBS at 4 °C. The organoids were permeabilized and blocked in a blocking solution (PBS + 0.5% (w/v) bovine serum albumin + 0.3% (v/v) Triton X-100) for 1 h at room temperature. Primary antibodies were diluted in 50 μl blocking solution and applied to organoids overnight with occasional mixing. After removal of the primary antibody solution, organoids were washed with 0.5 ml PBST (PBS + 0.3% Triton X-100) for 2 h and three times with occasional mixing. Secondary antibodies and DNA stains were diluted in 50 μl blocking solution overnight with occasional mixing. After removal of the secondary antibody solution, the organoids were washed with 0.5 ml PBST (for 2 h and three times) and finally stored in PBS. The primary antibodies used were rat anti-PODXL (R&D, MAB1556) at 1:40 dilution and mouse anti-ZO1 (Invitrogen, #33-9100) at 1:40 dilution. The secondary antibodies used were Alexa 568 anti-rat at 1:100 dilution and Alexa 647 anti-mouse at 1:100 dilution. The DNA stain was SYTOX Green (Invitrogen, S7020) used at 84 nM. Immunostained organoid samples were optically cleared using the second-generation ethyl cinnamate clearing protocol^50^.

### Fixed-sample imaging and 3D segmentation

Optically cleared organoids were transferred to an ethyl-cinnamate-resistant 96-well plate (Ibidi, #89621) for high-throughput imaging. The imaging was performed with an automated spinning-disc microscope (Yokogawa, Cell Voyager 7000S) equipped with a CSU-W1 spinning disc and a complementary metal–oxide–semiconductor camera (1,280 pixels × 1,080 pixels). To cover an entire well from a 96-well plate, four fields of view were acquired with a ×4 objective lens. These overview images were processed on the fly via a custom ImageJ macro executed by the SearchFirst module in the Wako Software Suite 2.0.20 that identified the coordinates of individual organoids. These coordinates were subsequently revisited with a ×20 air (NA = 0.75) Olympus objective. For each position, 300 planes with 0.8 μm spacing were acquired. Due to the ‘fish-bowl’ effect of ethyl cinnamate’s high refractive index (RI = 1.56), the effective *z* steps were 1.25 μm and the entire *z* stack encompassed 374 μm in sample depth. We used 2 × 2 binning on the camera, which resulted in images at 0.648 μm per pixel. For image analysis, we further binned the images in the *x*–*y* plane so that the 3D voxel dimensions were comparable (1.30 μm × 1.30 μm × 1.25 μm in the *x*, *y* and *z* directions). To segment the organoid outer boundary, we applied Otsu thresholding to the *z* stacks from the SYTOX Green channel. To segment the apical surface, we applied Otsu thresholding to the *z* stacks from the PODXL channel. The cell height of the epithelia was calculated for each organoid from identifying the boundaries from projections: cell height $h=\sqrt{{A}_{\mathrm{outer}}/(4\uppi )}-\sqrt{{A}_{\mathrm{inner}}/(4\uppi )}$, where *A*_outer_ and *A*_inner_ are the areas enclosed by the outer and inner (apical) convex hull boundaries, respectively. All the reported measurements have accounted for the effect of tissue shrinkage (factor of 0.603 in linear dimensions, experimentally determined) of the ethyl cinnamate clearing protocol.

### Live imaging and 3D segmentation

Neuroepithelial organoids were cultured as described above with the addition of 100 nM SiR-actin (Spirochrome) in the medium from day 1. Immediately after RA treatment on day 2, the organoids were transferred to custom multiwell chambers and imaged on a light sheet microscope (Viventis, LS1) equipped with a scientific complementary metal–oxide–semiconductor camera and 638 nm laser line. Every 30 min, *z* stacks were acquired at 3 µm intervals, covering a sample depth of 200 µm. For image analysis, we binned the images in the *x*–*y* plane so the voxel dimensions were nearly isotropic (2.76 μm × 2.76 μm × 3.00 μm in the *x*, *y* and *z* directions). To segment the organoid outer boundary, we applied multi-Otsu thresholding (classes = 3 using the lowest threshold) to the SiR-actin *z* stacks. To segment the apical surface, we applied multi-Otsu thresholding (classes = 4 using the highest threshold) to the SiR-actin *z* stacks.

### Surface construction and morphological analysis

The marching cubes algorithm was used to extract triangulated meshes from the segmented 3D images that represent the organoid outer boundary and apical surfaces. To reduce the mesh complexity, a combination of PyMesh functions were used to collapse the short edges (tolerance = 2 × the minimum voxel length of the input image), remove duplicated vertices, remove duplicated faces and remove degenerated/obtuse triangles. For each closed surface, we calculated volume *V*, surface area *A*, integral mean curvature *M* and Euler characteristic *χ* using the functions implemented in PyMesh and our custom code (Supplementary Note). These quantities were used to calculate the reduced volume $v=3\sqrt{4\uppi }\,V/{A}^{3/2}$, reduced curvature $m=M/\sqrt{4\uppi A\,}$, and topological genus *g* = 1 − *χ*/2 of epithelial lobules. Epithelial lobules with lumen volume *V* < 100 μm^3^ were excluded from the analysis. Organoid-level quantities were defined as total quantities *g* = ∑_*i*_*g*_*i*_ or as weighted averages 〈*v*〉 = ∑_*i*_*v*_*i*_*V*_*i*_/∑_*i*_*V*_*i*_ and 〈*m*〉 = ∑_*i*_*m*_*i*_*V*_*i*_/∑_*i*_*V*_*i*_, where index *i* enumerates all the *N* epithelial lobules of an organoid. All the computational analyses and visualizations in this study were performed using Python 3.7 with the libraries NumPy^51^, SciPy^52^, Pandas^53^, Matplotlib^54^, seaborn^55^, scikit-image^56^, PyMesh^57^, PyVista^58^ and Polyscope^59^.

### RNAseq and data processing

Organoids were grown in 35 mm MatTek dishes by seeding 3,000 5,000 R1 mouse ES cells in 50 μl Matrigel and cultured in 2 ml N2B27 medium at 37 °C, 5% CO_2_ via clonal growth as described in other work^60,61^. At day 2 (48 h post-seeding), the organoids were treated with or without 250 nM all-*trans* RA. The organoids were extracted from the Matrigel with a cell recovery solution (Corning 354253) at day 2 or at 8, 18, 30, 42, 54, 66 and 78 h after day 2. Three biological replicates were prepared for each cell population, yielding 45 independent samples for RNA extraction. For each sample, a minimum of 50,000 cells were collected for RNA extraction using the QIAGEN RNeasy Micro Kit. RNAseq libraries were prepared using the NEBNext Poly(A) mRNA Magnetic Isolation Module (E7490) and the NEBNext Ultra Directional RNA Library Prep Kit for Illumina (E7420) according to the manufacturer’s instructions. For ligation, custom adaptors were used (Adaptor-Oligo 1: 5’-ACA CTC TTT CCC TAC ACG ACG CTC TTC CGA TCT-3’; Adaptor-Oligo 2: 5’-P-GAT CGG AAG AGC ACA CGT CTG AAC TCC AGT CAC-3’). The libraries were sequenced as 75 bp single end on an Illumina NextSeq500 system. RNAseq reads were trimmed using Trim Galore v0.5.0, filtered to remove abundant sequences using Bowtie 2 v2.3.4.1, aligned to the GRCm38 genome (Ensembl release 94) using STAR v2.6.0c and summarized per gene with featureCounts (subread v1.6.2). Further analysis was performed using DESeq2 v1.18.1. To identify significantly differentially regulated genes between untreated versus RA-treated cell populations, we focused on the 18 and 30 h post-day 2 time points, and selected genes with log_2_ fold change less than –1 or greater than 1 and false discovery rate of 0.01 (based on adjusted *p* values) in both time points, identifying 569 upregulated and 250 downregulated genes.

### Reporting summary

Further information on research design is available in the Nature Research Reporting Summary linked to this article.

## Online content

Any methods, additional references, Nature Research reporting summaries, source data, extended data, supplementary information, acknowledgements, peer review information; details of author contributions and competing interests; and statements of data and code availability are available at 10.1038/s41567-022-01822-6.

## Supplementary information

Supplementary Information Supplementary note on theoretical analysis and figures.

Reporting Summary

Supplementary Video 1 Surface representations of an untreated organoid. Same organoid as in Fig. 1b. Organoid outer boundary (magenta) and apical surfaces (green) were reconstructed from day 4 organoids stained for DNA and anti-PODXL.

Supplementary Video 2 Surface representations of an RA-treated organoid. Same organoid as in Fig. 1b. Organoid outer boundary (magenta) and apical surfaces (green) were reconstructed from day 4 organoids stained for DNA and anti-PODXL.

Supplementary Video 3 Live imaging of an untreated organoid undergoing morphogenesis from day 2 to 4. Maximum intensity projections of SiR-actin volumetric images are shown as videos.

Supplementary Video 4 Live imaging of an RA-treated organoid undergoing morphogenesis from day 2 to 4. Maximum intensity projections of SiR-actin volumetric images are shown as videos.

Supplementary Video 5 Surface rendering of untreated organoid morphogenesis. This corresponds to SiR-actin (Supplementary Video 3). Organoid outer boundary (magenta, transparent) and apical surfaces (green).

Supplementary Video 6 Surface rendering of an RA-treated organoid morphogenesis. This corresponds to SiR-actin (Supplementary Video 4). Organoid outer boundary (magenta, transparent) and apical surfaces (green).

Supplementary Video 7 Mean curvature of an untreated organoid. The blue–red (low–high) colour map indicates the mean curvature of apical surfaces during day 2–4 morphogenesis of an untreated organoid.

Supplementary Video 8 Mean curvature of an RA-treated organoid. The blue–red (low–high) colour map indicates the mean curvature of apical surfaces during day 2–4 morphogenesis of an RA-treated organoid.

Supplementary Video 9 Gaussian curvature of an untreated organoid. The purple–green (low–high) colour map indicates the Gaussian curvature of apical surfaces during day 2–4 morphogenesis of an untreated organoid.

Supplementary Video 10 Gaussian curvature of an RA-treated organoid. The purple–green (low–high) colour map indicates the Gaussian curvature of apical surfaces during day 2–4 morphogenesis of an RA-treated organoid.

Supplementary Video 11 Surface representations of an organoid treated with 2 μM HA130. Same organoids as in Fig. 3d. Organoid outer boundary (magenta) and apical surfaces (green) were reconstructed from day 4 organoids stained for DNA and anti-PODXL.

Supplementary Video 12 Surface representations of an organoid treated with RA and 2 μM HA130. Same organoids as in Fig. 3d. Organoid outer boundary (magenta) and apical surfaces (green) were reconstructed from day 4 organoids stained for DNA and anti-PODXL.

Supplementary Table S1 Genes that are differentially regulated in response to RA treatment as identified from the RNAseq of organoids. Columns represent (1) gene symbol, (2) log_2_ fold change in the transcript, RA-treated over untreated organoids, (3) –log_10_(*p*_adj_) and (4) *p*_adj_, where *p*_adj_ is the adjusted *p* value of statistical significance as determined by DESeq2 for three biological replicates of untreated organoids versus three biological replicates of RA-treated organoids from 18 and 30 h post-day 2 time points.

## Data Availability

The RNAseq data associated with this study have been deposited to NCBI GEO under accession code GSE214368. All other data that support the plots within this paper and other findings of this study are available from the corresponding authors upon reasonable request. Source data are provided with this paper.

## Source data

Source Data Fig. 1 Source data for the plots in Fig. 1.
Source Data Fig. 2 Source data for the plots in Fig. 2.
Source Data Fig. 3 Source data for the plots in Fig. 3.
Source Data Extended Data Fig. 7 Source data for the plots in Extended Data Fig. 7.

## Code availability

Codes to analyse the data and perform numerical calculations are available upon reasonable request.
