# Supplementary material for: Topological morphogenesis of neuroepithelial organoids
Source: Nat Phys. 2022 Nov 21;19(2):177–83. doi: 10.1038/s41567-022-01822-6 (PMC9928582; doi:10.1038/s41567-022-01822-6)
Supplement: Supplementary file 1 — Supplementary note on theoretical analysis and figures. [file 41567_2022_1822_MOESM1_ESM.pdf]

nature physics

Letter https://doi.org/10.1038/s41567-022-01822-6

Topological morphogenesis of neuroepithelial organoids

In the format provided by the authors and unedited

Supplementary Note

Topological morphogenesis of neuroepithelial organoids

Keisuke Ishihara[1,2,3,4,5,†], Arghyadip Mukherjee[1,2,3,4,†], Elena Gromberg[5], Jan Brugués[1,2,3,4,*], Elly M. Tanaka[5,*], Frank Jülicher[2,3,4,*]

[1]Max Planck Institute of Molecular Cell Biology and Genetics (MPI-CBG); 01307 Dresden, Germany.

[2]Max Planck Institute for the Physics of Complex Systems (MPI-PKS); 01187 Dresden, Germany.

[3]Center for Systems Biology Dresden; 01307 Dresden, Germany.

[4]Cluster of Excellence Physics of Life, TU Dresden; 01062 Dresden, Germany.

[5]Research Institute of Molecular Pathology (IMP), Campus-Vienna-Biocenter 1, 1030 Vienna, Austria.

[†]These authors contributed equally to this work.

[*]Corresponding authors: brugues@mpi-cbg.de, elly.tanaka@imp.ac.at, julicher@pks.mpg.de

Contents

1 Morphological analysis of epithelial lobules and organoids **2**
1.1 Integral geometry of smooth surfaces . . . 2
1.2 Integral geometry of discretized surfaces . . . 3
1.3 Morphological analysis with shape diagrams . . . 4
1.3.1 Spherocylinder . . . 4
1.3.2 Wiffle ball . . . 5

2 Topological transitions in a system of fluid surfaces **7**
2.1 Energetics of topological transitions . . . 7
2.2 Elastic moduli of epithelial tissues and their relation to cell mechanics . . . 8
2.3 Morphogenesis guided by *trans* fusion . . . 9

1 Morphological analysis of epithelial lobules and organoids

Here we provide a brief summary of geometric concepts used in the analysis of epithelial lobules, and an extended discussion on the geometric theory of epithelial surfaces.

1.1 Integral geometry of smooth surfaces

We consider the apical surface of the epithelial lobules as closed smooth surfaces. At any point on the surface, the curvature is captured by the mean curvature

$$H = \frac{1}{2}(C_1 + C_2) \tag{1}$$

and the Gaussian curvature

$$K = C_1 C_2 \quad , \tag{2}$$

where C_1, C_2 are the principal curvatures (Fig. 1a).

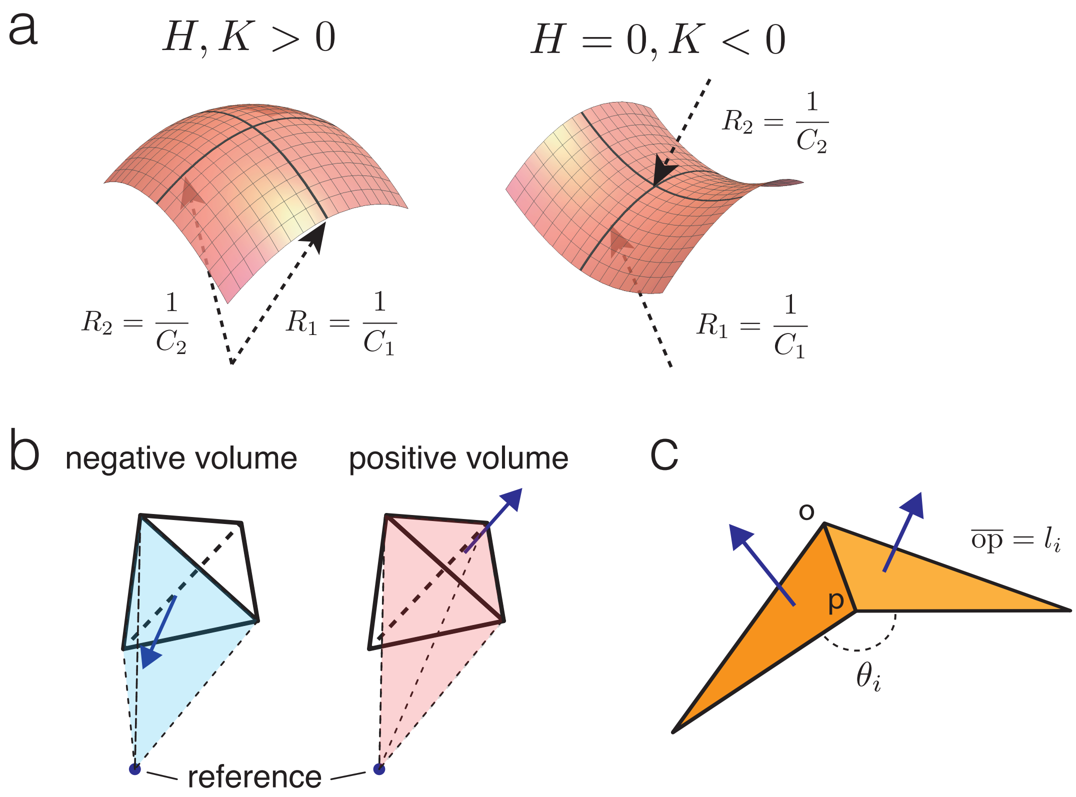

Fig. SN1: **a**, Principle curvatures C_1 and C_2 and associated radii of curvature R_1 and R_2 are shown for a convex (left) and saddle (right) surface. Mean curvature H and Gaussian curvature K are indicated. **b**, Signed volumes (negative or positive) are indicated for depicted tetrahedrons based on the position of the reference point. **c**, Dihedral angle θ_i between two adjacent faces is shown, the associated edge $\overline{\text{op}}$ has a length of l_i.

Integral geometry provides a set of “global” morphological descriptors called Minkowski functionals that characterize the geometry and topology of objects (*1*). In 3-dimensional space, they are

$$\text{Volume:} \quad V = \int dV \tag{3}$$

$$\text{Surface Area:} \quad A = \int dA \tag{4}$$

$$\text{Integral Mean Curvature:} \quad M = \int H dA \tag{5}$$

$$\text{Euler characteristic:} \quad \chi = \frac{1}{2\pi} \int K dA = 2(N - g) \quad , \tag{6}$$

where dV and dA are the volume and area element, respectively. Topology is captured by the Euler characteristic χ of the object, which relates to the Gaussian curvature via the Gauss-Bonnet theorem $2\pi\chi = \int K dA$. For a single object, $\chi = 2 - 2g$ where g is the topological genus that quantifies the number of handles. For an epithelial organoid consisting of multiple epithelial lobules, $\chi = 2(N - g)$ where N is the number of distinct lobules and g is total genus.

1.2 Integral geometry of discretized surfaces

Volumetric images of organoids are segmented and used to construct triangular meshes to represent the apical surfaces of epithelial lobules. Thus, we use discrete approximations of the Minkowski functionals Eq.(3-6) to characterize geometry and topology. The meshes are defined as polyhedron, with N_v number of vertices connected via N_e number of edges, and encapsulate N_f number of flat faces. The surface area A and volume V then are calculated as

$$A = \sum_i^{N_f} A_i \quad , \tag{7}$$

and

$$V = \sum_i^{N_f} V_{\uparrow,i} \quad , \tag{8}$$

where A_i is the face area of the ith face and $V_{\uparrow,i}$ is the signed volume of a tetrahedron defined by the three vertices of the face and an arbitrary reference point (Fig. 1b). The sign of $V_{\uparrow,i}$ is determined by asking if the face normal vector is pointing towards (negative volume) or away (positive volume) from the reference point.

For the integral mean curvature M, we follow Steiner's approach (*2*) to mollify the polyhedron, or smoothen its edges and vertices, with a ball of radius $\epsilon > 0$. In the limit of very small ϵ, we obtain

$$M = \sum_i^{N_e} \frac{1}{2} \theta_i l_i \quad , \tag{9}$$

where l_i is the edge length and θ_i is the dihedral angle at the edge ((*3*), Fig. 1c).

The Euler characteristic χ of the polyhedron is calculated using Euler's formula,

$$\chi = N_v - N_e + N_f \quad . \tag{10}$$

1.3 Morphological analysis with shape diagrams

We use two non-dimensional metrics, reduced volume v and reduced curvature m to capture the shapes of epithelial lobules,

$$\text{Reduced volume:} \quad v \equiv 3\sqrt{4\pi}\frac{V}{A^{3/2}} \tag{11}$$

$$\text{Reduced curvature:} \quad m \equiv \frac{M}{\sqrt{4\pi A}} \quad . \tag{12}$$

v is an isoperimetric quantity that captures the compactness of a surface in three-dimensions, while m measures the curvature-area mismatch. For a sphere, both v and m are equal to unity. In the following section, we analyze idealized shapes of spherocylinders and wiffle balls with shape diagrams (v, m).

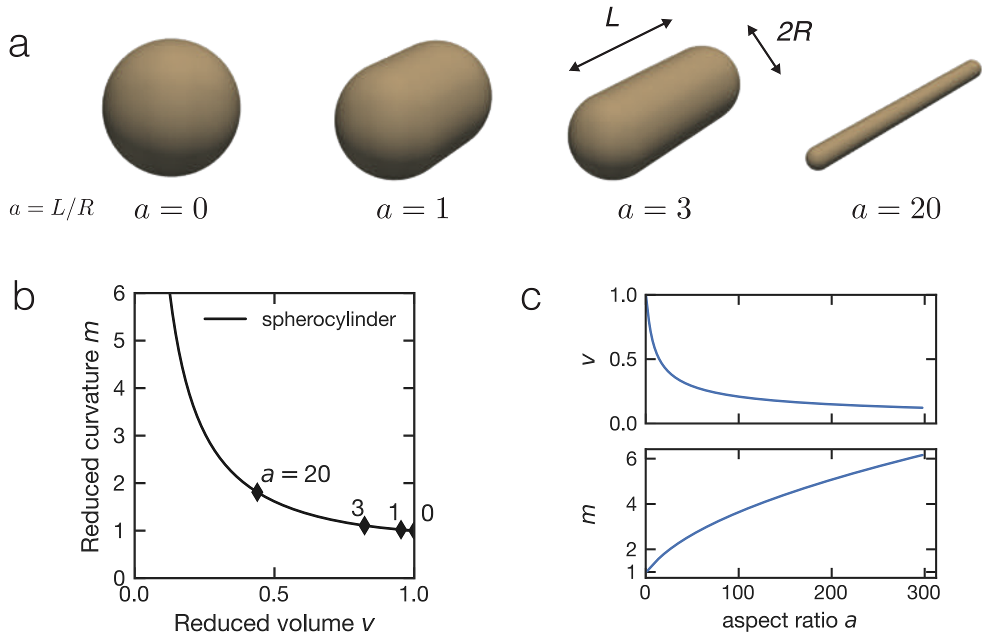

Fig. SN2: **a**, Spherocylinders of increasing (left to right) aspect ratio a are shown. **b**, Spherocylinders with specific values of a are represented as points in the shape diagram. The solid-line represents the parametric curve for the spherocylinder family. **c**, Reduce volume v (top) and reduced curvature m (bottom) are shown as a function of aspect ratio a.

1.3.1 Spherocylinder

During morphogenesis, neuroepithelial lobules start from spherical shapes and become increasingly elongated tubular structures. The spherocylinder provides an idealised model to investigate this experimentally observed shape transition. A spherocylinder consists of a cylinder of length L with spherical caps at both ends, where the radius of the cylinder and of the spheres are both R (Fig. 2a). This shape can be parametrized by the aspect ratio $a = L/R$. For aspect ratio $a = 0$, we recover a sphere. For higher values of aspect ratio a, the spherocylinder represents increasingly elongated tubes. The Minkowski functionals are then given by,

$$V = \pi R^2 L + \frac{4\pi}{3} R^3 = \pi R^3 (a + \frac{4}{3}) \tag{13}$$
$$A = 2\pi R L + 4\pi R^2 = 2\pi R^2 (2 + a) \tag{14}$$
$$M = \frac{1}{2R} \cdot 2\pi R L + \frac{1}{R} \cdot 4\pi R^2 = \pi R (a + 4) \tag{15}$$
$$\chi = 2 \tag{16}$$

The reduced volume v and reduced curvature m can be parametrized with the aspect ratio a as,

$$v = \frac{3a/4 + 1}{(1 + a/2)^{3/2}}, \tag{17}$$
$$m = \frac{a/4 + 1}{\sqrt{1 + a/2}} \quad . \tag{18}$$

These equations define a characteristic curve for spherocylinder in the shape diagram (Fig. 2b,c).

1.3.2 Wiffle ball

The final geometry of the Day 4 neuroepithelial lobules resemble a wiffle ball, a familiar children's toy in North America (Fig. 3b). We consider two concentric spheres with radii R and $R + d$, connected via p number of passages (see Fig. 3,a-b). For simplicity we consider all identical passages with central aperture 2θ (Fig. 3c). We model the passages with the inner surface of a torus with major radius $r = (R+d/2)\sin\theta$ and minor radius $d/2$.

We obtain $\theta_{\max}(p)$ using approximate solutions for the optimal packing of p points on a spherical surface (*4*). To a good approximation $\theta_{\max}(p) \sim \sqrt{\pi/p}$. The minima of the aperture angle $\theta_{\min}(d/R)$ is set by the condition $r > d/2$.

Note that for a wiffle ball with p passages the genus $g = p - 1$. The Minkowski functionals are given by,

$$V = \frac{4\pi - p\,\Omega_p}{3} \left((R + d)^3 - R^3\right) + p\,V_p \tag{19}$$
$$A = (4\pi - p\,\Omega_p) \left((R + d)^2 + R^2\right) + p\,A_p \tag{20}$$
$$M = (4\pi - p\,\Omega_p) d + p M_p \tag{21}$$
$$\chi = 2 - 2g = 2 - 2(p - 1) = 4 - 2p \quad . \tag{22}$$

Here V_p, A_p, M_p and Ω_p are respectively the volume, surface area, integral mean curvature, and the solid angle of one passage,

$$V_p = \frac{\pi}{2} \left(\frac{d}{2}\right)^2 \cdot 2\pi \left(r - \frac{4}{3\pi}\frac{d}{2}\cos\theta\right) = \frac{\pi^2}{4} d^2 r - \frac{\pi}{6} d^3 \cos\theta \tag{23}$$
$$A_p = \pi d \int_{\theta-\pi}^{\theta} d\theta' \left(r + \frac{d}{2}\sin\theta'\right) = \pi^2 d r - \pi d^2 \cos\theta \tag{24}$$
$$M_p = \pi d \int_{\theta-\pi}^{\theta} d\theta' \left(r + \frac{d}{2}\sin\theta'\right) \left(\frac{2}{d} + \frac{\sin\theta'}{r + \frac{d}{2}\sin\theta'}\right) = 2\pi^2 r - 4\pi d \cos\theta \tag{25}$$
$$\Omega_p = \int d\Omega = \int_0^{\theta} d\theta' 2\pi \sin\theta' d\theta' = 2\pi(1 - \cos\theta) \quad . \tag{26}$$

We find that for $d/R << 1$, to a leading order $v \sim d/R$. The variation in reduced curvature m arises from dependencies on θ and p, which to a linear order can be approximated to,

$$m \sim \sqrt{\frac{\pi^3}{2}\theta p} - \sqrt{2\pi}\,\frac{d}{R}(p-1) \quad . \tag{27}$$

As a result, the parametric curve for a wiffle ball in the shape diagram is almost a vertical line (Fig. 3d, e).

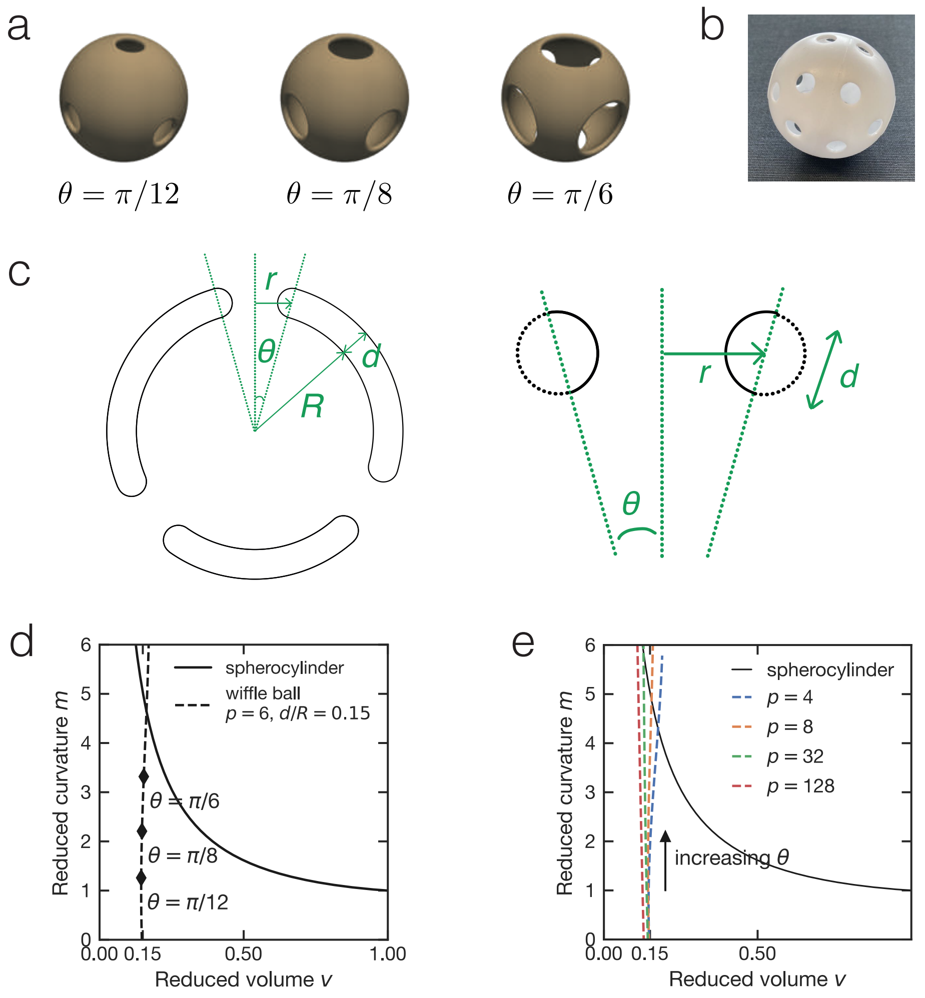

Fig. SN3: **a**, Wiffle balls with $p = 6$ passages are shown for varying opening angle θ. **b**, Image showing a wiffle ball, a familiar children's toy in North America. **c**, Left, cross-sectional diagram of a wiffle ball depicting geometric variables; thickness d, inner radius R and opening angle θ. Right, cross-sectional view of a passage of the wiffle ball; geometric variables are indicated. **d**, Parametric curve (dashed line) for a wiffle ball with $p = 6$ passages is shown for $d/R = 0.15$. **e**, Parametric curve (colored dashed lines) for a wiffle ball with varying number of passages (see legend) is shown with $d/R = 0.15$.

2 Topological transitions in a system of fluid surfaces

In this section, we discuss topological transitions in a system of fluid surfaces with N closed surfaces or lobules and g number of handles. The transition in topology of the system is thus characterized by changes in the topological indices N and g. The mechanics of such fluid surfaces can be captured with a bending energy

$$E_b = \int \left(\kappa H^2 + \bar{\kappa} K\right) dA \quad , \tag{28}$$

where H , K an dA respectively denote the local mean curvature, the Gaussian curvature of the surface, and the area element. The bending rigidity κ and the Gaussian rigidity $\bar{\kappa}$ are elastic moduli that capture the resistance of the shape to bending and saddle-splay deformations, respectively. Due to the Gauss-Bonnet theorem, $\int K dA = 2\pi\chi = 4\pi(N - g)$ is a topological invariant and depends only on the number of surfaces N and total genus g. The Gaussian rigidity $\bar{\kappa}$ hence describes the resistance to topological changes that occur via changes in N as well as g. The bending rigidity κ dictates changes in shape, but also changes in N.

2.1 Energetics of topological transitions

Motivated by the morphology of the organoids observed at the early time points, let us first consider an ensemble of spherical lobules. For any sphere of radius R, the mean curvature $H = 1/R$, Gaussian curvature $K = 1/R^2$ and the bending energy $E_b = 4\pi(\kappa + \bar{\kappa})$. For a system of N spheres of any size

$$E_b = 4\pi(\kappa + \bar{\kappa})N \tag{29}$$

$$\implies \Delta E_b = 4\pi(\kappa + \bar{\kappa})\Delta N \quad . \tag{30}$$

In such a system, a decrease in N ($\Delta N < 0$) reduces E_b iff $\kappa + \bar{\kappa} > 0$. The change in bending energy for a *trans* fusion is $\Delta E_b \simeq -4\pi(\kappa + \bar{\kappa})$ (see Fig. SN4a), and hence is favored also when $\kappa + \bar{\kappa} > 0$ (Fig. SN4b, dashed line). This criteria hence determines the conditions for *trans* fusion and stability of a system of spherical lobules. On the other hand, high genus shapes are favored when $\bar{\kappa} > 0$ and *cis* fusion lowers bending energy by $\Delta E_b \simeq -4\pi\bar{\kappa}$ (Fig. 4a). Together for a system of N lobules with g handles the change in bending energy due to topological transitions can be represented as,

$$\Delta E_b \simeq 4\pi(\kappa + \bar{\kappa})\Delta N - 4\pi\bar{\kappa}\Delta g \tag{31}$$

$$\implies \frac{\Delta E_b}{4\pi\kappa} \simeq \left(1 + \frac{\bar{\kappa}}{\kappa}\right)\Delta N - \frac{\bar{\kappa}}{\kappa}\Delta g \quad . \tag{32}$$

Using these energetic criteria, we propose a simple two dimensional state diagram (Fig. SN4b) for a system whose initial state consists of multiple spheres. We predict three morphological outcomes, depending on which mode of topological transitions are favored. We can further simplify this state diagram for $\kappa > 0$ into a one-dimensional state diagram as a function of the reduced Gaussian rigidity $\bar{\kappa}/\kappa$ (see Main text and Fig. 3b).

Resultant morphologies can then be interpreted by studying the variation of only one parameter, $\bar{\kappa}/\kappa$. High genus structures lie in region III, where $\bar{\kappa}/\kappa > 0$. A system of spheres and tubes, formed via *trans* fusion, lie in region II for which $\bar{\kappa}/\kappa > -1$.

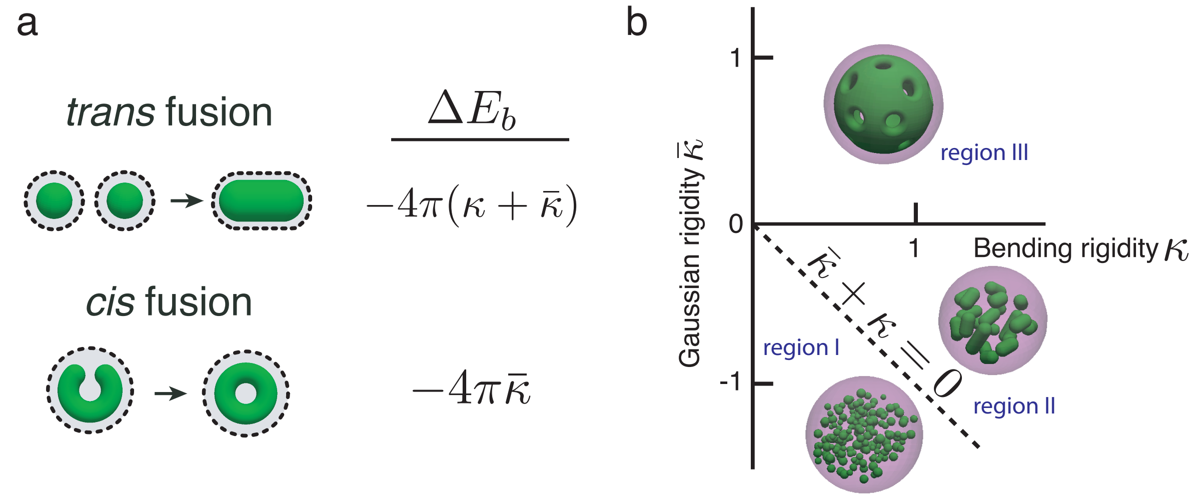

Fig. SN4: **a**, Changes in bending energy ΔE_b is shown for examples of *trans* (top) and *cis* (bottom) fusion. **b**, A state diagram representing three regions. In region I (below dashed line), fusions are disfavored, while in region II only *trans* fusion is favored. Both *trans* and *cis* fusion are favored in region III giving rise to structures with many passages. Insets show example systems with representative topology.

More generally for any system of N lobules and g handles ($g-1$ passages)

$$E_b = 4\pi N(\kappa s_l + \bar{\kappa}) - 4\pi\bar{\kappa}g \quad , \tag{33}$$

where $s_l = (\int H^2 dA)/N$ is a number that captures the average shape energy of the lobules. Note that for a system of spheres $s_l = 1$ and it is shown by Willmore that $s_l \geq 1$ (*5*). In that case the condition for *trans* fusion being favored yields a correction $\bar{\kappa}/\kappa > -s_l$. This modified criteria is naturally satisfied when $\bar{\kappa}/\kappa > -1$ as $s_l \geq 1$, hence for simplicity we limit our discussion to $s_l \sim 1$.

2.2 Elastic moduli of epithelial tissues and their relation to cell mechanics

Epithelial tissues are active complex materials, whose mechanical properties emerge from the multi-scale interactions of molecules and cells. The bending rigidity of an epithelium has been estimated to be $\kappa \sim 10^{-12} J$ for mono-layered, spherical cysts (*6*) consistent with scaling predictions (*7*). However, how the bending and Gaussian rigidity of an epithelium relates to cellular mechanics and geometry is an open question. Several groups have presented mechanical models of tissue deformations in 3 dimensions that are accessible to qualitative interpretation (*8–12*). For example, Rozman and colleagues (*11*) consider a 3d vertex model of an epithelium that consists of identically shaped cells of fixed volume V_c, characterised by the apical, basal and lateral tensions, T_a, T_b, T_l, respectively. Through a coarse graining procedure, the effective tissue-scale bending and Gaussian rigidities were obtained as

$$\kappa = \frac{1}{2}\left(\frac{T_a+T_b}{T_l}\right)^{1/3} T_l V_c^{2/3} \quad , \quad \bar{\kappa} = \frac{1}{3}\left(\frac{T_a+T_b}{T_l}\right)^{1/3}\left(\left(\frac{T_a+T_b}{T_l}\right)^2 - \frac{3}{4}\right) T_l V_c^{2/3} \quad . \tag{34}$$

Here these expressions have been derived for equilibrium cell height $h = ((T_a+T_b)/T_l)^{2/3}$.

Using these expressions, we write the reduced Gaussian rigidity as

$$\frac{\bar{\kappa}}{\kappa} = \frac{2}{3}\left(\left(\frac{T_a + T_b}{T_l}\right)^2 - \frac{3}{4}\right) \quad . \tag{35}$$

This suggests that a decrease in cellular tension $T_a + T_b$ leads to a decrease in reduced Gaussian rigidity, which could lead to an overall reduction of epithelial fusion events. In the neuroepithelial organoids the actomyosion cortex is predominantly located at the apical side, indicating that $T_a > T_b, T_l$ and hence T_a would be more affected by a change in actomyosin activity. This is supported by our experiments in neuroepithelial organoids, where we reduce cell contractility using the ROCK kinase inhibitor Y-27632 and observe a large number of lobules with few passages (Extended Fig. 7).

2.3 Morphogenesis guided by *trans* fusion

In this section we discuss how successive *trans* fusion of almost spherical lobules can give rise to tubular shapes and as a result drive the morphological trajectory of neuroepithelial organoids. To discuss this we use the idealized geometric model of spherocylinder (section 1.3.1), which in the limit of vanishing aspect ratio ($a = 0$) is equivalent to a sphere.

Consider the *trans* fusion of two spheres of equal radii R_0, where the total surface area and volume remain unchanged by the fusion event. This geometric constraint, then determines the geometry after fusion. The area and the volume of the final shape is then given by

$$A = 8\pi R_0^2 \quad , \quad V = \frac{8}{3}\pi R_0^3 \quad . \tag{36}$$

The reduced volume v for this final shape is thus $v = 1/\sqrt{2} \simeq 0.707$ whereas for a sphere $v = 1$. As a result the fused lobule does not stay spherical. A perturbative analysis of fluid surfaces near the spherical state shows that prolate structures are more stable than oblate structures (*13*). Such prolate shapes are well represented by the spherocylinder family. The shape of a spherocylinder of radius R and length L formed by coaslescence of n identical spheres of radius R_0 is characterized then by its aspect ratio a, which is a function of n. The aspect ratio $a(n)$ can be obtained by solving the area and volume constraint given by

$$4\pi R^2 + 2\pi RL = n4\pi R_0^2 \quad , \quad \frac{4}{3}\pi R^3 + \pi R^2 L = n\frac{4}{3}\pi R_0^3 \quad . \tag{37}$$

The exact solution of $a(n)$ can be obtained (see Fig. 5a, blue solid line). To a good numerical approximation $a(n) \sim (2/\sqrt{\pi})\sqrt{n-1} + (13/3)(n-1)$ (see Fig. 5a , yellow dashed line).

We find that the aspect ratio a to be a monotonic function of n, hence implying how progressive trans fusion of spherical lobules can give rise to elongate tubes with increasing aspect ratio. This will result in decreasing reduced volume v and increasing reduced curvature m (see Fig. SN5b,c), depicting a trajectory in (v, m) space similar to observed morphogenetic trajectory of neuroepithelial organoids (see Fig. 2a).

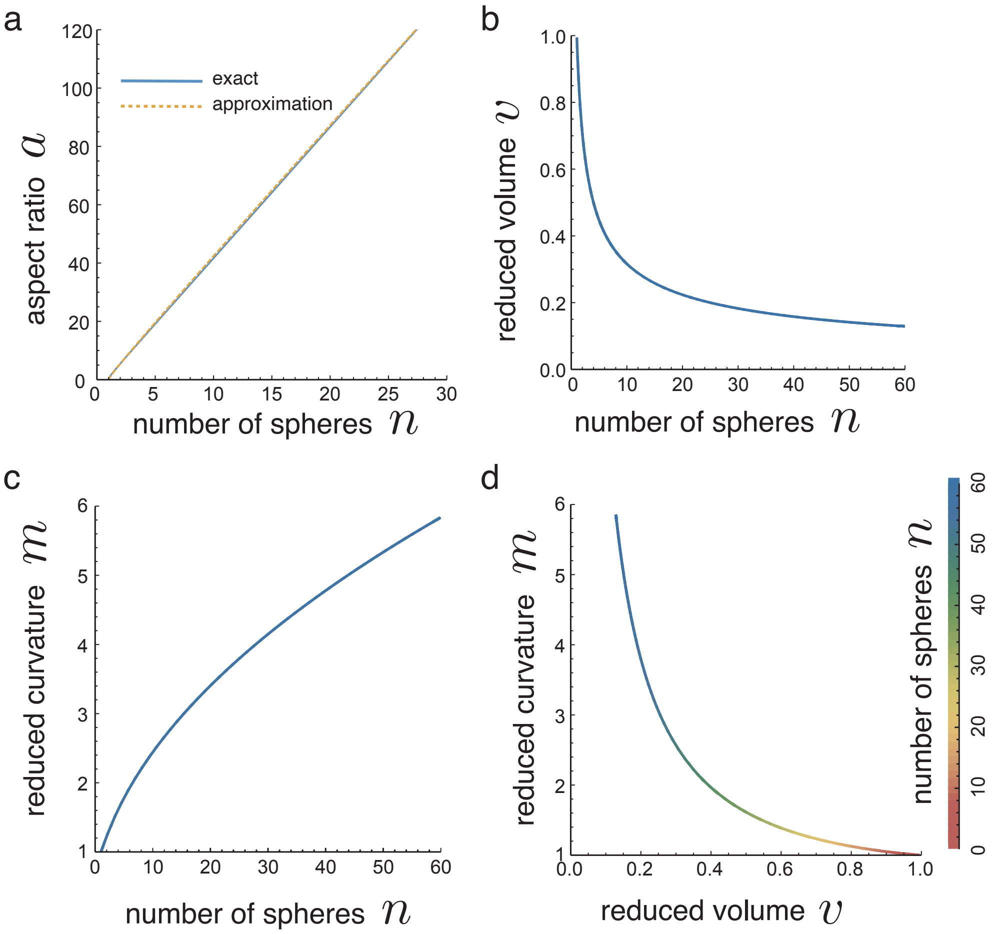

Fig. SN6: **a**, Aspect ratio a of a spherocylinder formed by coalescence of n identical spheres is shown as a function of n. The approximate solution (yellow dashed line, see text) lies closely to the exact solution (blue solid line) for $a(n)$. **b**,**c**, Reduced volume v and reduced curvature m of the said spherocylinder is shown as a function of n. **d**, Parametric curve depicting morphogenetic trajectory of a spherocylinder in (v, m) space parametrized by n.

References

1. Klaus R Mecke. Additivity, Convexity, and Beyond: Applications of Minkowski Functionals in Statistical Physics. In *Statistical Physics and Spatial Statistics*, pages 111–184. Springer, Berlin, Heidelberg, Berlin, Heidelberg, 2000.
2. Jakob Steiner. Über parallele flächen. *Monatsber. Preuss. Akad. Wiss*, 2:114–118, 1840.
3. Frank Jülicher. The morphology of vesicles of higher topological genus: conformal degeneracy and conformal modes. *Journal de physique II*, 6(12):1797–1824, 1996.
4. NJA Sloane, R Hardin, and Smith WD. Tables of Spherical Codes. NeilSloane.com/packings.

5. Thomas Willmore. *Total curvature in Riemannian geometry.* Ellis Horwood, 1982.

6. Anastasiya Trushko, Ilaria Di Meglio, Aziza Merzouki, Carles Blanch-Mercader, Shada Abuhattum, Jochen Guck, Kevin Alessandri, Pierre Nassoy, Karsten Kruse, Bastien Chopard, et al. Buckling of an epithelium growing under spherical confinement. *Developmental cell*, 54(5):655–668, 2020.

7. Andrew R Harris, Loic Peter, Julien Bellis, Buzz Baum, Alexandre J Kabla, and Guillaume T Charras. Characterizing the mechanics of cultured cell monolayers. *Proceedings of the National Academy of Sciences*, 109(41):16449–16454, 2012.

8. Edouard Hannezo, Jacques Prost, and Jean-Francois Joanny. Theory of epithelial sheet morphology in three dimensions. *Proceedings of the National Academy of Sciences*, 111(1):27–32, 2014.

9. Nebojsa Murisic, Vincent Hakim, Ioannis G Kevrekidis, Stanislav Y Shvartsman, and Basile Audoly. From discrete to continuum models of three-dimensional deformations in epithelial sheets. *Biophysical journal*, 109(1):154–163, 2015.

10. Christina Bielmeier, Silvanus Alt, Vanessa Weichselberger, Marco La Fortezza, Hartmann Harz, Frank Jülicher, Guillaume Salbreux, and Anne-Kathrin Classen. Interface contractility between differently fated cells drives cell elimination and cyst formation. *Current Biology*, 26(5):563–574, 2016.

11. Jan Rozman, Matej Krajnc, and Primož Ziherl. Collective cell mechanics of epithelial shells with organoid-like morphologies. *Nature communications*, 11(1):1–9, 2020.

12. Vincent F Fiore, Matej Krajnc, Felipe Garcia Quiroz, John Levorse, H Amalia Pasolli, Stanislav Y Shvartsman, and Elaine Fuchs. Mechanics of a multilayer epithelium instruct tumour architecture and function. *Nature*, 585(7825):433–439, 2020.

13. Udo Seifert, Karin Berndl, and Reinhard Lipowsky. Shape transformations of vesicles: Phase diagram for spontaneous-curvature and bilayer-coupling models. *Physical review A*, 44(2):1182, 1991.
