# Supplementary material for: Topological morphogenesis of neuroepithelial organoids
Source: Nat Phys. 2022 Nov 21;19(2):177–83. doi: 10.1038/s41567-022-01822-6 (PMC9928582; doi:10.1038/s41567-022-01822-6)
Supplement: Supplementary file 2 — Reporting Summary [file 41567_2022_1822_MOESM2_ESM.pdf]

nature portfolio

Corresponding author(s): Jan Brugues

Last updated by author(s): Sep 30, 2022

Reporting Summary

Nature Portfolio wishes to improve the reproducibility of the work that we publish. This form provides structure for consistency and transparency in reporting. For further information on Nature Portfolio policies, see our Editorial Policies and the Editorial Policy Checklist.

Statistics

For all statistical analyses, confirm that the following items are present in the figure legend, table legend, main text, or Methods section.

n/a	Confirmed	
☐	☒	The exact sample size (*n*) for each experimental group/condition, given as a discrete number and unit of measurement
☐	☒	A statement on whether measurements were taken from distinct samples or whether the same sample was measured repeatedly
☐	☒	The statistical test(s) used AND whether they are one- or two-sided<br>*Only common tests should be described solely by name; describe more complex techniques in the Methods section.*
☒	☐	A description of all covariates tested
☒	☐	A description of any assumptions or corrections, such as tests of normality and adjustment for multiple comparisons
☐	☒	A full description of the statistical parameters including central tendency (e.g. means) or other basic estimates (e.g. regression coefficient) AND variation (e.g. standard deviation) or associated estimates of uncertainty (e.g. confidence intervals)
☒	☐	For null hypothesis testing, the test statistic (e.g. *F*, *t*, *r*) with confidence intervals, effect sizes, degrees of freedom and *P* value noted<br>*Give P values as exact values whenever suitable.*
☒	☐	For Bayesian analysis, information on the choice of priors and Markov chain Monte Carlo settings
☒	☐	For hierarchical and complex designs, identification of the appropriate level for tests and full reporting of outcomes
☒	☐	Estimates of effect sizes (e.g. Cohen's *d*, Pearson's *r*), indicating how they were calculated

Our web collection on statistics for biologists contains articles on many of the points above.

Software and code

Policy information about availability of computer code

Data collection	Software for operating Viventis LS1 and Yokogawa CV7000 microscopes. Both are commercial software.
Data analysis	Python (with packages Matplotlib, Seaborn, Scikit-Image, PyMesh, PyVista, and Polyscope).

For manuscripts utilizing custom algorithms or software that are central to the research but not yet described in published literature, software must be made available to editors and reviewers. We strongly encourage code deposition in a community repository (e.g. GitHub). See the Nature Portfolio guidelines for submitting code & software for further information.

Data

Policy information about availability of data

All manuscripts must include a data availability statement. This statement should provide the following information, where applicable:

- Accession codes, unique identifiers, or web links for publicly available datasets
- A description of any restrictions on data availability
- For clinical datasets or third party data, please ensure that the statement adheres to our policy

Source data are available for this paper. The RNAseq data associated with this study has been deposited to NCBI GEO under the accession code GSE214368.
All other data that support the plots within this paper and other findings of this study are available from the corresponding author upon reasonable request.

Human research participants

Policy information about studies involving human research participants and Sex and Gender in Research.

Reporting on sex and gender	N.A.
Population characteristics	N.A.
Recruitment	N.A.
Ethics oversight	N.A.

Note that full information on the approval of the study protocol must also be provided in the manuscript.

Field-specific reporting

Please select the one below that is the best fit for your research. If you are not sure, read the appropriate sections before making your selection.

☒ Life sciences ☐ Behavioural & social sciences ☐ Ecological, evolutionary & environmental sciences

For a reference copy of the document with all sections, see nature.com/documents/nr-reporting-summary-flat.pdf

Life sciences study design

All studies must disclose on these points even when the disclosure is negative.

Sample size	No sample size calculation was performed. We assayed quantitative trends as a function of experimental time and drug concentration. The main conclusions of the paper are supported with the presented sample size.
Data exclusions	Organoid samples that resulted in poor image segmentation (resulting as outliers in fractional lumen volume) were excluded.
Replication	We combined 3D morphological measurements from different organoid samples, which were similar to each other, hence replicability is ensured.
Randomization	Not applicable as we assayed quantitative trends as a function of experimental time and drug concentration. We did not observe covariation of metrics with respect to position in a 96 well plate.
Blinding	Not applicable as we assayed quantitative trends as a function of experimental time and drug concentration. The same parameters for automated image analysis were applied for the same imaging experiments.

Reporting for specific materials, systems and methods

We require information from authors about some types of materials, experimental systems and methods used in many studies. Here, indicate whether each material, system or method listed is relevant to your study. If you are not sure if a list item applies to your research, read the appropriate section before selecting a response.

Materials & experimental systems

n/a	Involved in the study	
☐	☒	Antibodies
☐	☒	Eukaryotic cell lines
☒	☐	Palaeontology and archaeology
☒	☐	Animals and other organisms
☒	☐	Clinical data
☒	☐	Dual use research of concern

Methods

n/a	Involved in the study	
☒	☐	ChIP-seq
☒	☐	Flow cytometry
☒	☐	MRI-based neuroimaging

Antibodies

Antibodies used	Mouse anti-ZO1 (Invitrogen, Clone ZO1-1A12, #33-9100), Rat anti-PODXL (R&D, Clone # 192703, MAB1556)
Validation	Invitrogen verified the ZO1 antibody in a knock down experiment. The authors have verified the PODXL antibody using knock out cell lines.

Eukaryotic cell lines

Policy information about cell lines and Sex and Gender in Research

Cell line source(s)	E14 mouse embryonic stem cells (a gift from Marta Shahbazi, MRC Laboratory of Molecular Biology); R1 mouse embryonic stem cells (a gift from Ronald Naumann, MPI-CBG).
Authentication	None of the cell lines used were authenticated.
Mycoplasma contamination	All cell lines tested negative for mycoplasma contamination.
Commonly misidentified lines (See ICLAC register)	none
